# Feruloyl esterase-producing lactobacillus screening and its synergistic effect with homolactic and heterolactic bacteria on corn stover silage

**DOI:** 10.3389/fmicb.2026.1755745

**Published:** 2026-03-06

**Authors:** Lin Wang, Zilu Cai, Fusheng Li, Zhuang Ke, Ran Tao, Bin Ye, Cansheng Yuan, Qin He

**Affiliations:** 1 College of Rural Revitalization, Jiangsu Open University, Nanjing, China; 2 Sanya Institute of Nanjing Agricultural University, Department of Microbiology, College of Life Sciences, Nanjing Agricultural University, Key Lab of Microbiological Engineering of Agricultural Environment, Ministry of Agriculture, Nanjing, China; 3 Institute of Chemical Industry of Forest Products, Chinese Academy of Forestry, Nanjing, China; 4 Institute of Microbe and Host Health, Linyi University, Linyi, China

**Keywords:** corn stover silage, ferulic acid esterase-producing lactobacillus, heterofermentation, homofermentation, lactic acid bacteria, silage additives

## Abstract

**Introduction:**

This study investigated the synergistic effects of combining ferulic acid esterase (FAE)-producing lactobacillus with homofermentative and heterofermentative lactic acid bacteria (LAB) on the fermentation quality, nutrient composition, and aerobic stability of corn stover silage.

**Methods:**

In this study, five LAB strains were isolated and identified from various silages. Among them, strain AR1 was identified as *Lactiplantibacillus pentosus* and exhibited high FAE activity. The homofermentative strains R10, JF1, and JF2 were identified as *Lactiplantibacillus plantarum*, *Pediococcus acidilactici*, and *Pediococcus pentosaceus*, respectively. The heterofermentative strain R3 was Leuconostoc mesenteroides subsp. mesenteroides. A total of 11 treatment groups were designed in triplicate, including a control group (CK), a commercial inoculant group (JCK), and nine LAB treatments at three concentrations (1 × 10^6^, 1 × 10^7^, 1 × 10^8^ CFU/g FW). The groups were AR1-only (R), a homofermentative-heterofermentative combination (LPL), and a homofermentative-heterofermentative combination with AR1 (LPLR).

**Results:**

The results showed that the co-fermentation of homofermentative and heterofermentative strains improved silage fermentation quality. The addition of AR1 to the combination of homofermentative and heterofermentative LAB further enhanced lactic acid and acetic acid production, decreased neutral and acid detergent fiber contents, and improved aerobic stability. Principal component analysis and membership function analysis identified the LPLR group (an equal mixture of AR1, R10, JF2, and R3 at 1 × 10^7^ CFU/g fresh weight) as the optimal formula, achieving the highest comprehensive score of 0.696. **Discussion:** This study provides a theoretical basis for the development of silage additives.

## Introduction

As a microbial-driven process, ensiling effectively preserves the nutritional quality and extends the storage period of fresh forages, while also serving as a promising pretreatment method for enhancing sustainable biomass utilization (Liu et al., 2019). Corn stover is one of the most abundant and renewable biomass resources, which has become an attractive substrate for ensilage. Consequently, corn stover silage has been considered a potential approach for producing high-quality feed. However, the utilization of corn stover is constrained by its rigid cell wall structure composed of cellulose, hemicellulose, and lignin (Fang et al., 2023). The structure affects fiber digestion and nutrient absorption in ruminants *in vivo* (Guo et al., 2022). Ensiling enhances the digestibility of corn stover, prolongs its storage, and improves its nutrition and antioxidant properties by lactobacillus fermentation (He et al., 2020).

Natural silage is inherently unstable and often fails to ensure consistent feed quality. The application of additives could effectively inhibit the growth of harmful microorganisms, enhance nutritional quality, increase fiber degradation efficiency, and improve both fermentation quality and aerobic stability of the silage (Muck et al., 2018; Huang et al., 2024). In particular, lactic acid bacteria (LAB) and enzymes are the most widely used in silage production (Okoye et al., 2023a; Lynch et al., 2015). For LAB silage, homofermentative LAB dominate at the early stage of silage by rapidly lowering pH, whereas heterofermentative LAB prolong preservation by acetic acid production. For instance, Zhang et al. (2021) reported that the homofermenters *Lactiplantibacillus plantarum* and *Pediococcus pentosaceus* improved silage nutritional quality by producing lactic acid, while the heterofermenter *Lactobacillus buchneri* enhanced aerobic stability by acetic acid production in corn silage. Consequently, the combined application of these strains balances rapid acidification with long-term stability (Muck et al., 2018). Furthermore, previous research showed that the co-inoculation of heterofermentative (*L. buchneri*) and homofermentative (*L. plantarum*) LAB synergistically reduced nutrient degradation and enhanced aerobic stability (Jin et al., 2024). In addition to microbial inoculants, enzymes (e.g., cellulase and hemicellulase) are widely used to degrade neutral detergent fiber (NDF) and acid detergent fiber (ADF) in plants, thereby improving nutrient digestibility in silage. For example, studies reported that the addition of lignocellulolytic enzymes, such as ferulic acid esterase and laccase, enhanced the structural accessibility of cellulose by disrupting the plant cell wall (Kumar et al., 2022; Addah et al., 2012). This structural disruption facilitates the release of fermentable carbohydrates from the plant, providing additional substrates to LAB (Addah et al., 2016). However, the practical application and synergistic efficiency of exogenous enzymes may be limited in silage. Therefore, recent research has focused on selecting novel lignocellulose-degrading LAB strains capable of endogenous enzyme production for sustainable silage production.

The ferulic acid ester bond is a key cross-linking bond between hemicellulose and lignin in the plant cell wall. Ferulic acid esterase (FAE) catalyzes the cleavage of this ester bond, thereby promoting the separation of hemicellulose and lignin, and facilitating the degradation of cellulose by other enzymes such as cellulase and hemicellulose (Addah et al., 2012). Most of the FAE-producing microorganisms reported are fungi, such as *Aspergillus niger*, *Penicillium chrysogenum*, *Fusarium oxysporum*, and *Neurospora crassa* (Benoit et al., 2006; Pérez-Rodríguez et al., 2016). However, the co-fermentation of the fungi during ensiling would lead to mycotoxin production, affect aerobic stability, increase nutrient loss, and inhibit LAB fermentation. These adverse effects could lead to silage spoilage and thus pose a risk to animal health (Wang et al., 2021). Consequently, screening FAE-producing LAB for silage is an effective method to overcome these problems. Several FAE-producing LAB strains were identified, including *Lactiplantibacillus plantarum*, *Lactobacillus acidophilus*, and *Limosilactobacillus fermentum* (Oliveira et al., 2019; Xu et al., 2020). Silage inoculation with these strains improved silage quality and digestibility, while modulating the rumen microbial communities to enhance feed utilization (Li et al., 2023). However, the synergistic effects of FAE-producing lactobacillus, homolactic and heterolactic bacteria on corn stover silage are not clear.

In this study, homofermentative, heterofermentative, and FAE-producing LAB were screened from silage to evaluate their synergistic effects on corn stover silage. The purpose of this study was to investigate the synergistic effects of FAE-producing lactobacillus, homofermentative and heterofermentative LAB on silage, optimize bacterial combinations, and provide a theoretical basis for the development of microbial silage additives.

## Materials and methods

### Isolation and identification of homolactic and heterolactic bacteria, and FAE-producing LAB

The samples utilized in this experiment for the isolation of LAB were collected from corn stover silage, rapeseed silage, and alfalfa silage in Liuhe District, Nanjing, China in October 2022. They were collected using sterile sampling bags, stored at 4°C, and subjected to the isolation and purification steps for LAB upon arrival at the laboratory. LAB were isolated and purified according to the methods described by Yang et al. (2016). The sample (10 g) evenly mixed with 90 mL sterile water. The mixture serially diluted from 10^–1^ to 10^–9^ using sterilized water, and spread 200 μL of each dilution on the surface of de Man, Rogosa, Sharpe (MRS) agar containing 1% CaCO_3_. The plates were incubated upside down under the anaerobic condition at 37°C for 72 h. After incubation, LAB colonies that exhibited calcium dissolution circles were selected and purified.

The purified single colonies were picked into MRS broth, and incubated at 37°C, 150 rpm/min for 48 h. The cultures were then diluted with physiological saline to 10^8^ colony forming units (CFU)/mL as bacterial suspensions. These suspensions were inoculated into MRS broth at a 5% (v/v) inoculation amount and incubated at 37°C for 24 h. During this period, samples were taken every 2 h to measure the optical density at 600 nm (OD_600_) and the pH value. Morphological, physiological and biochemical tests of LAB and Gram-staining responses were examined after 24 h of incubation on MRS agar. Catalase activity was determined using the method of Yang et al. (2016). The experimental methods of salt tolerance, acid resistance, and temperature resistance were the same as Yang et al. (2016). Carbohydrate assimilation and fermentation of 49 compounds with one control were identified on API 50 CH strips (bioMérieux, Lyon, France).

The FAE-producing LAB was preliminarily screened by MRS agar with 0.1% (v/v) methyl ferulate as the only carbon source (Ding et al., 2019). Furthermore, we further selected the highest FAE-producing LAB through detecting their FAE activity of all the candidate strains. The FAE activity was determined according to the method of Wu et al. (2021). FAE activity (1 U) was defined as the enzymes required to release 1 μmol ferulic acid per min by degrading methyl ferulate at 30°C. The feruloyl esterase activity was detected using an Ultimate 3,000 high-performance liquid chromatography (HPLC) system (Thermo Fisher Scientific, United States) equipped with a C_18_ reverse phase chromatography column (4.6 × 250 mm, 5 μm) and a UV detector. The mobile phase was the mixture of methanol with glacial acetic acid (28:72) at a flow rate of 0.6 mL/min under the column temperature of 40°C, and the detection wavelength was 320 nm.

### DNA amplification, sequencing, and phylogenetic analysis

The nearly full-length 16S rDNA gene was amplified by polymerase chain reaction (PCR) using universal bacterial primers 27F (5’-AGAGTTTGATCCTGGCTCAG-3’) and 1492R (5’-GGCTACCTTGTTACGACTT-3’). The PCR reaction mixture comprised 25 μL 2 × Rapid Taq Master Mix (Vazyme Biotech Co., Ltd., Nanjing, China), 1 μL Template DNA, 1 μL each primer (10 μM), and 22 μL ddH_2_O. The PCR reaction conditions were consistent with the method of Wu et al. (2021). After PCR amplification, the products were detected by agarose gel electrophoresis. Successfully amplified products were sequenced by Sangon Biotech Co., Ltd. (Shanghai, China). The identity of the isolates was determined by nucleotide-nucleotide BLAST against the DNA Database of Japan (DDBJ) database, and phylogenetic trees were constructed using MEGA 11.0. The nucleic acid sequences of the isolates were submitted to the DDBJ database.

### Effect of FAE-producing LAB addition on corn stover silage fermented by homolactic and heterolactic bacteria

Corn stover was obtained from Liuhe District, Nanjing City, China. The stover was harvested in grain maturation, air-dried to a moisture content below 70%, and chopped into 2–4 cm pieces. The materials were stored in polyethylene plastic bags, with 250 g in each bag, and the bags were vacuum sealed. After activating, the screened LAB was transferred into MRS broth at a 5% (v/v) inoculation amount and cultured at 37°C, 120 rpm/min for 24 h. The cultures were centrifuged at 6,000 rpm/min for 3 min. The supernatants were discarded. The bacterial cells were collected and diluted with sterile saline to 1 × 10^8^ CFU/mL.

Based on pre-experimental results, the optimal homolactic and heterolactic bacteria formulation was identified as LPL, comprising equal amounts of *Lactiplantibacillus plantarum* R10 (LC909651), *Pediococcus pentosaceus* JF2 (LC909649), and *Leuconostoc mesenteroides* subsp. *mesenteroides* R3 (LC909650). This study investigated the effects of an FAE-producing *Lactiplantibacillus pentosus* AR1 (LC909652) adding to the LPL group on fermentation quality, nutritional composition, and aerobic stability. A total of 11 treatment groups were evaluated in triplicate. The CK group was sprayed with sterile saline, and the JCK group was treated with a commercial inoculant (Henan Nongfukang Biotechnology Co., Ltd., Zhengzhou, China) at a concentration of 5 × 10^6^ CFU/g fresh weight (FW). The other nine groups comprised three LAB treatments with each at three different concentrations of low (1 × 10^6^ CFU/g FW), medium (1 × 10^7^ CFU/g FW), and high (1 × 10^8^ CFU/g FW). The R group contained solely *L. pentosus* AR1. The LPL group comprised *L. plantarum* R10, *P. pentosaceus* JF2, and *Leuc. mesenteroides* R3 (1:1:1). The LPLR group consisted of *L. plantarum* R10, *P. pentosaceus* JF2, *Leuc. mesenteroides* R3, and *L. pentosus* AR1 (1:1:1:1). The additives for all groups were applied at the same total volume of 5 mL/kg FW. Each sample was fermented at 25°C for 60 d.

### Analysis of chemical composition, microbial populations, and fermentation quality

To prepare silage extract, 10 g silage fermented for 60 d was taken and placed in 90 mL distilled water. The mixture was extracted at 4°C for 24 h. The acidity of the silage was measured at room temperature using a pH meter. Samples were dried at 65°C for 48 h to determine dry matter (DM). The water-soluble carbohydrate (WSC) content was assayed by the anthrone-sulfuric acid colorimetry (Grandy et al., 2000). According to Wang et al. (2024), the determination of lactic acid (LA), acetic acid (AA), butyric acid (BA), and propionic acid (PA) was performed using the Ultimate 3,000 HPLC system (Thermo Fisher Scientific, United States). The mobile phase was 3 mmol/L HClO_4_. A C_18_ reverse phase column was employed, maintained at 50°C. The flow rate was set at 1 mL/min, and the detection wavelength was 210 nm. An injection volume of 20 μL was utilized. The crude protein (CP) content was analyzed using a fully automated Kjeldahl nitrogen analyzer (FOSS Kjeltec 2300). The contents of NDF and ADF were determined by an ANKOM A2000I fiber analyzer (ANKOM, A2000i, United States). The ash content was measured gravimetrically after incineration, and hemicellulose (HC) content was calculated based on the difference between NDF and ADF. The ammonia nitrogen (AN) concentration was determined using the phenol-sodium hypochlorite method (Kleinschmit et al., 2005). The counts of LAB, molds, yeasts, and aerobic bacteria (AB) were determined as described by Zielińska et al. (2015). AB were counted on Luria-Bertani agar, while yeasts and molds were counted on Potato Dextrose Agar.

### Aerobic stability

After 60 d of ensiling, a 250 g silage sample from each treatment was transferred into a 500 mL plastic bucket. The bucket was covered with gauze to prevent contamination. The samples were maintained under the controlled environmental condition at 25 ± 1°C. The sensor of the temperature recorder (model MDL-1048A, Shanghai Tianhe Automation Instrument Co., Ltd.) was positioned in the center of the silage block, and the temperature was recorded every 4 h for 168 h. Aerobic stability was determined as the duration required for the silage temperature to consistently exceed the ambient temperature by 2°C (Moraes et al., 2023). The pH value and the content of LA and AN, as well as the counts of LAB, yeast, and AB, were measured at 3 and 7 d of aerobic exposure. The entire experiment was terminated after 7 d of aerobic exposure.

### Comprehensive evaluation of silage

The fermentation characteristics, nutrient composition, and aerobic stability of different addition amount and additive combinations were evaluated using principal component analysis (PCA) (Xu et al., 2024). Based on the PCA results, the fermentation conditions, chemical composition, and aerobic stability of corn stover silage were evaluated using the fuzzy mathematical membership function method (Li J. et al., 2022). Positive indicators included WSC, CP, DM, LA, PA, and AA after 60 d of silage fermentation, as well as LA and LAB count after 7 d of aerobic exposure. Negative indicators were ADF, NDF, pH, HC, AN, and ash, after 60 d of fermentation and AN, AB count, pH, and yeast count after 7 d of aerobic exposure. Average membership values were calculated from these indicators and utilized to rank the treatment groups. A higher average value indicates a better silage quality.

The calculation formula was as follows:


$$U_{X+}=\frac{X_{i\,j}-X_{i\,m\,i\,n}}{X_{i\,m\,a\,x}-X_{i\,m\,i\,n}}$$ (1)

$$U_{X-}=1-\frac{X_{i\,j}-X_{i\,m\,i\,n}}{X_{i\,m\,a\,x}-X_{i\,m\,i\,n}}$$ (2)

$${\bar{U}}_{X}=\frac{1}{\text{n}}\,\overset{n}{\underset{j=1}{\sum }}U_{X}$$ (3)

*U_X+_* is the membership function value for positive indicators (Equation 1), whereas *U_X–_* is the membership function value for negative indicators (Equation 2). *X_ij_* represents the actual measured value of each indicator, while *X_imax_* and _X*imin*_ represent the maximum and minimum values, respectively, among all measured data. *i* represents each treatment, *j* represents the silage indicator, and *n* represents the total number of indicators (Equation 3).

### Statistical analysis

Data were recorded in Microsoft Excel (Microsoft Corp., Redmond, WA, United States) and checked for normality and homogeneity of variance. The data were analyzed by one-way analysis of variance (ANOVA) to evaluate the effects of LAB inoculants and inoculation concentration in SPSS 26.0 (IBM Corp., Armonk, NY, United States). Duncan’s test was used to determine significant differences between means. Differences were considered significant when *p* < 0.05. The results were presented as mean ± standard deviation (SD). The principal component analysis was performed using OriginPro^®^ 2021b software (OriginLab, Washington, United States).

## Results

### Isolation and identification of LAB strains and their biological characteristics

**FIGURE 1:**
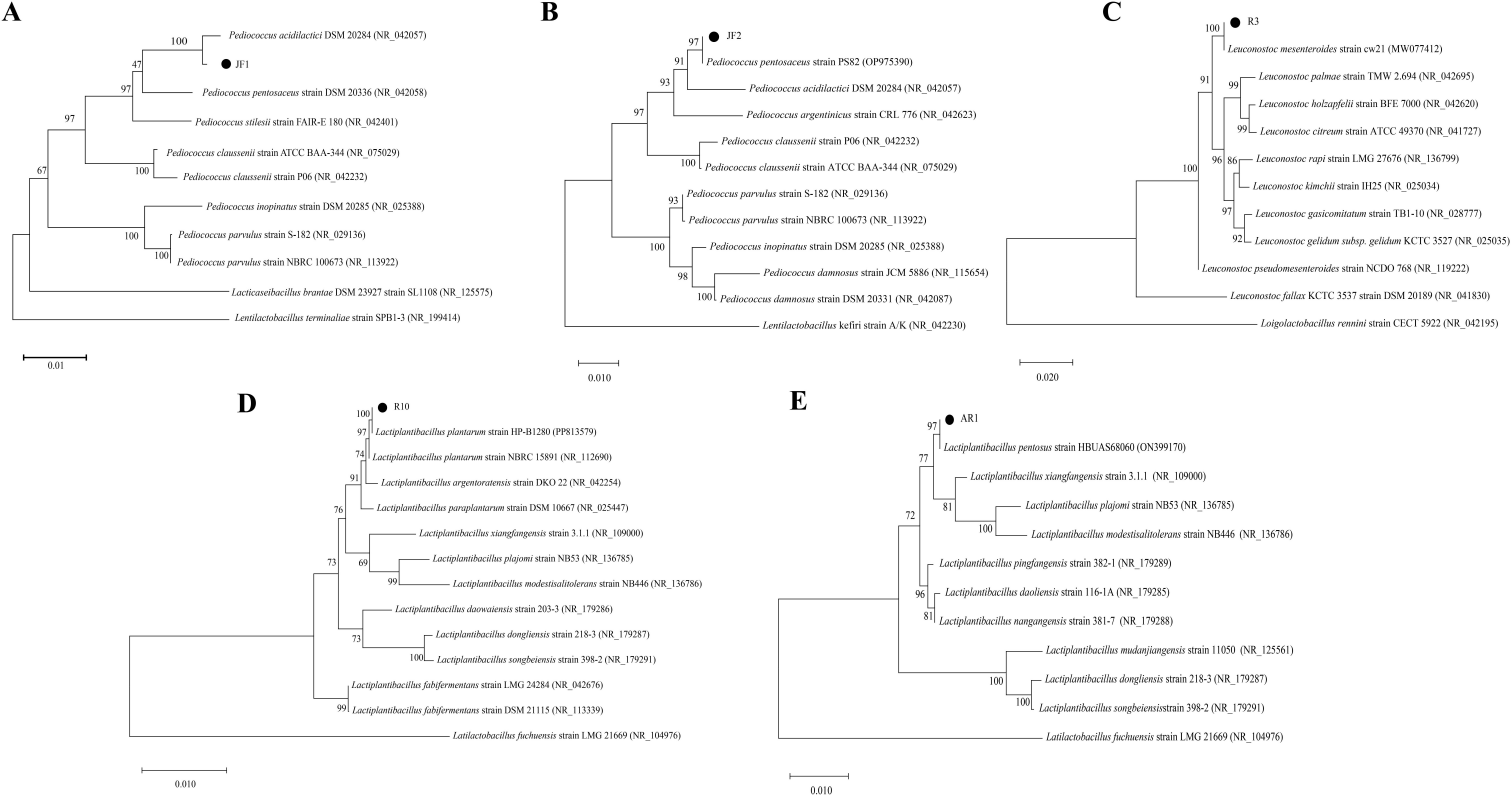
Phylogenetic trees of the LAB isolates. **(A)** JF1; **(B)** JF2; **(C)** R3; **(D)** R10; **(E)** AR1.

A total of 52, 16, and 21 strains were isolated from corn stover, alfalfa, and rapeseed silages, respectively, and identified as LAB based on morphological, physiological, and 16S rDNA sequencing analyses. It was found that strains JF1, JF2, R3, and R10, based on evaluating biological indicators such as growth rate, acid production rate, temperature tolerance, acid resistance, and salt tolerance. These strains exhibited faster growth and acid production rates, stronger environmental tolerance, and met the requirements for LAB used in ensiling. Furthermore, a FAE-producing lactobacillus strain (AR1) was isolated from these silages, and its feruloyl esterase activity reached 7.11 ± 0.58 mU/mL. Morphologically, the five LAB strains displayed round, creamy white or off-white colonies with a raised center, moist, and smooth surfaces, ranging from 1 to 5 mm in diameter. All strains were able to ferment sucrose, xylose, galactose, raffinose, melibiose, arabinose, maltose, glucose, fructose, cellobiose, and mannose, while JF1 did not ferment sorbitol. Additionally, R3 lacked the ability to ferment sorbose. Strains JF1, JF2, R10, and AR1 were homofermentative, while strain R3 was heterofermentative. R10 and AR1 were Gram-positive rods, whereas the other strains were Gram-positive cocci. All strains were catalase-negative and glucose-negative (Table 1). According to 16S rDNA gene sequence analysis, these five isolated strains were identified as *Pediococcus acidilactici* (JF1), *Pediococcus pentosaceus* (JF2), *Leuconostoc mesenteroides* subsp. *mesenteroides* (R3), *Lactiplantibacillus plantarum* (R10), and *Lactiplantibacillus pentosus* (AR1), respectively (Figure 1).

**TABLE 1:** Phenotypic and biochemical characteristics of the isolated strains.

Item	Strain				
	JF1	JF2	R3	R10	AR1
Fermentation type	Homogeneous	Homogeneous	Heteromorphic	Homogeneous	Homogeneous
FAE activity (mU/mL)	-	-	-	-	7.11 ± 0.58
Shape	Coccus	Coccus	Coccus	Rod	Rod
Sucrose	+	+	+	+	+
Xylose	+	+	+	+	+
Trehalose	+	+	+	+	+
Sorbose	+	+	-	+	+
Ribose	+	+	+	+	+
Galactose	+	+	+	+	+
Raffinose	+	+	+	+	+
Melibiose	+	+	+	+	+
Sorbitol	-	+	+	+	+
Arabinose	+	+	+	+	+
Rhamnose	-	-	-	+	-
Maltose	+	+	+	+	+
Lactose	+	+	+	-	+
Glucose	+	+	+	+	+
Fructose	+	+	+	+	+
Cellobiose	+	+	+	+	+
Mannitol	-	+	+	-	+
Mannose	+	+	+	+	+
Gluconate	-	-	-	-	-
Reduction of nitrate	-	-	-	-	-
Catalase test	-	-	-	-	-
Gram’s stain	+	+	+	+	+

FAE, ferulic acid esterase. +, positive; -, negative.

**FIGURE 2:**
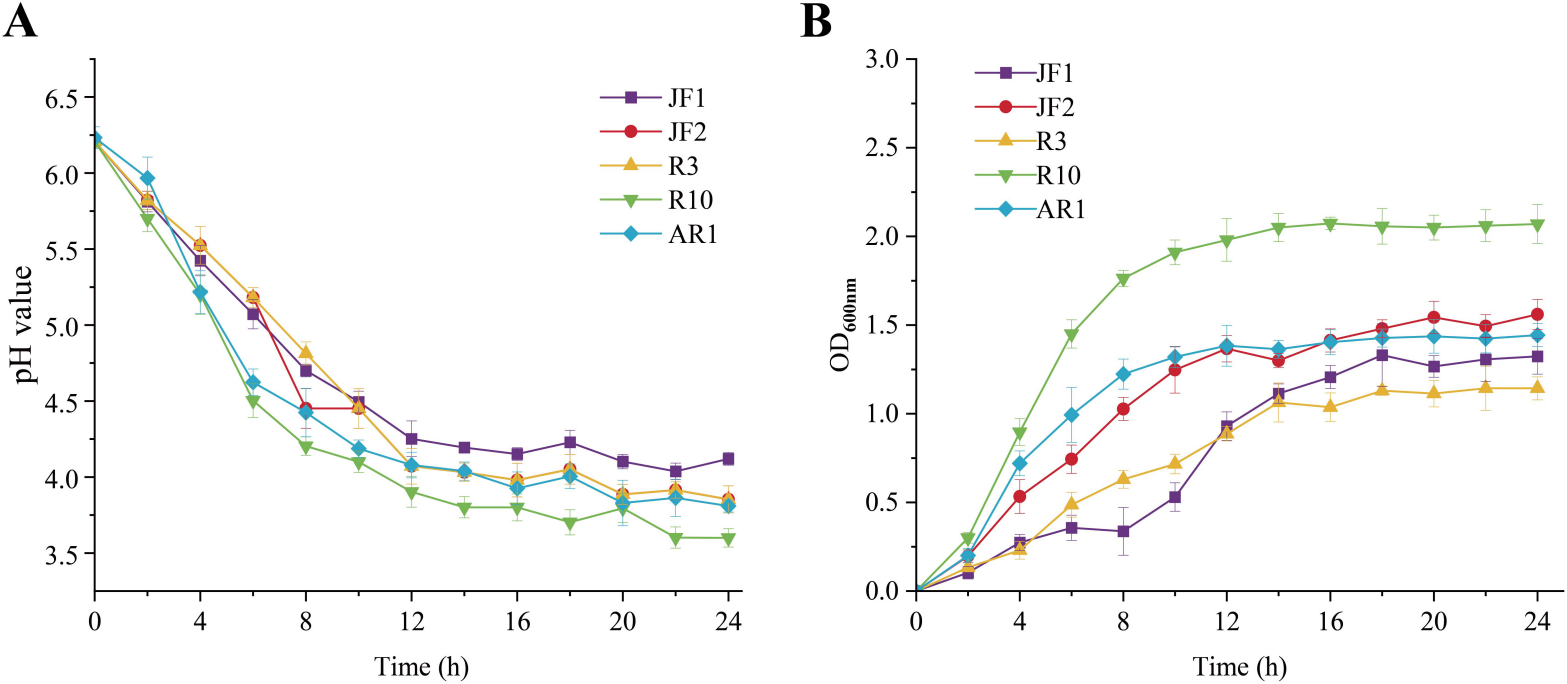
pH curves and growth curves of the LAB isolates. **(A)** pH curves of the LAB isolates. **(B)** Growth curves of the LAB isolates.

The acid-producing ability of LAB is a key determinant of silage fermentation quality. As shown in Figure 2A, the five isolated LAB strains demonstrated a rapid pH decrease when cultured in MRS broth. The pH began to decline after 2 h of incubation and exhibited a significant drop by 8 h, reaching levels below 5.0 for all strains except strain R3. By 24 h, the pH for all the strains had decreased below 4.5. Due to the inhibition of bacterial metabolism by the acidic environment, the pH stabilized at their minimum levels. Figure 2B showed the microbial growth curves of the isolates. All strains reached a growth phase by 14 h. Strain R10 exhibited the fastest growth between 2 and 10 h, achieving the highest density among the strains (*p* < 0.05). The rapid microbial growth was consistent with the highest acid concentration produced in Figure 2A.

### Synergistic effect of homofermentative and heterofermentative LAB on corn stover silage and aerobic stability

Selected homofermentative LAB strains, including *L. plantarum* (LP), *P. pentosaceus* (PP), and *P. acidilactici* (PA), and the heterofermentative strain *Leuc. mesenteroides* (LM), were applied to silage additives. The pre-experimental treatments were designated as LM, LPA (LP+PA), LPP (LP+PP), LAL (LP+PA +LM), and LPL (LP+PP+LM). During the ensiling fermentation, all additive groups significantly enhanced the comprehensive quality of corn stover silage (Supplementary Table 1). This was mainly manifested by a significant reduction in pH, AN, and fiber content of the feed (*p* < 0.05). The content of LA and DM showed a significant increase (*p* < 0.05). There was no significant difference in CP content between the LAB treatments and the CK (*p* > 0.05). The additive group with heterofermentative LAB produced significantly higher AA than the CK and other groups (*p* < 0.05). Homofermentative LAB groups had better fermentation quality and higher acid production, whereas the group treated solely with homofermentative LAB exhibited poorer aerobic stability. Conversely, the LM group showed weaker acid production but greater aerobic stability (Supplementary Table 2). In addition, there was a significant difference in ash content between LPA and LM group (*p* < 0.05). The results of the subordination function analysis indicated that the average membership value of the combination of LPL (*L. plantarum*, *P. pentosaceus*, and *Leuc. mesenteroides*) was the highest (0.72), resulting in better ensiling effects (Supplementary Table 3).

### Effect of FAE-producing LAB addition on fermentation characteristics and microbial counts of corn stover silage

Based on pre-experimental results, the optimal homolactic and heterolactic bacteria formulation was identified as LPL. This study investigated the synergistic effects of adding the FAE-producing strain AR1 to LPL group on fermentation quality. The results of fermentation characteristics and microbial counts were displayed in Table 2. All inoculated groups exhibited significantly lower pH value compared to the CK (*p* < 0.05). The lowest pH was the medium concentration LPL group (3.58), which did not differ significantly from the JCK group (3.61). At low and high concentrations, LPL and LPLR exhibited stronger acidification capacity than R groups. However, there was no significant difference between the R group and the LPLR group at medium concentration (*p* > 0.05). The phenomenon indicated that adding FAE-producing LAB did not further enhance the acidification efficiency of LPL.

**TABLE 2:** Effects of different inoculants and inoculation levels on fermentation quality of corn stover silage after 60 d of ensiling.

Item	Addition amount	Group				
		CK	JCK	R	LPL	LPLR
pH	Low	3.89 ± 0.13^a^	3.61 ± 0.07^e^	3.77 ± 0.11^c^	3.67 ± 0.12^d^	3.68 ± 0.12^d^
	Medium			3.72 ± 0.08^cd^	3.58 ± 0.07^e^	3.63 ± 0.05^d^
	High			3.83 ± 0.02^b^	3.63 ± 0.18^d^	3.73 ± 0.13^cd^
WSC (%)	Low	6.64 ± 0.85^a^	6.39 ± 0.77^b^	6.07 ± 0.49^d^	6.19 ± 0.38^c^	6.43 ± 0.68^b^
	Medium			5.86 ± 0.67^e^	6.23 ± 0.77^c^	6.15 ± 0.43^cd^
	High			5.96 ± 0.48^de^	6.04 ± 0.53^de^	5.93 ± 0.79^de^
LA (%)	Low	7.37 ± 0.86^e^	8.89 ± 0.51^a^	7.98 ± 0.32^d^	8.42 ± 0.65^c^	8.23 ± 0.84^cd^
	Medium			8.09 ± 0.59^d^	8.83 ± 0.49^a^	8.78 ± 0.96^ab^
	High			8.25 ± 0.47^c^	8.73 ± 0.58^b^	7.87 ± 0.69^de^
AA (%)	Low	2.23 ± 0.45^cd^	2.25 ± 0.58^c^	1.98 ± 0.29^d^	2.17 ± 0.74^cd^	2.33 ± 0.65^c^
	Medium			2.32 ± 0.45^c^	2.58 ± 0.64^b^	2.97 ± 0.18^a^
	High			2.22 ± 0.47^c^	2.83 ± 0.25^a^	2.52 ± 0.37^b^
BA (%)	Low	ND	ND	ND	ND	ND
	Medium			ND	ND	ND
	High			ND	ND	ND
PA (%)	Low	ND	ND	ND	ND	ND
	Medium			ND	ND	ND
	High			ND	ND	ND
LAB (log_10_ cfu/g FW)	Low	7.38 ± 0.52^e^	8.67 ± 0.61^a^	8.21 ± 0.35^c^	8.16 ± 0.49^d^	8.72 ± 0.68^a^
	Medium			8.64 ± 0.58^a^	8.28 ± 0.73^c^	8.67 ± 0.38^a^
	High			8.37 ± 0.19^b^	8.74 ± 0.65^a^	8.15 ± 1.01^d^
AB (log_10_ cfu/g FW)	Low	5.21 ± 0.37^a^	4.04 ± 0.23^d^	4.98 ± 0.35^ab^	4.38 ± 0.59^cd^	4.58 ± 0.72^c^
	Medium			4.67 ± 0.71^bc^	4.27 ± 0.79^cd^	4.07 ± 1.32^d^
	High			4.45 ± 0.58^c^	4.83 ± 1.23^b^	4.16 ± 0.68^d^
Yeasts (log_10_ cfu/g FW)	Low	<2.00	<2.00	<2.00	<2.00	<2.00
	Medium			<2.00	<2.00	<2.00
	High			<2.00	<2.00	<2.00
Molds (log_10_ cfu/g FW)	Low	<2.00	<2.00	<2.00	<2.00	<2.00
	Medium			<2.00	<2.00	<2.00
	High			<2.00	<2.00	<2.00

FW, fresh weight; WSC, water soluble carbohydrate; LA, lactic acid; AA, acetic acid; BA, butyric acid; PA, propanoic acid; LAB, lactic acid bacteria; AB, aerobic bacteria; ND, not detected. JCK stands for commercial microbial agent control group; R stands for *Lactiplantibacillus pentosus* alone; LPL stands for combination of *Lactiplantibacillus plantarum*, *Pediococcus pentosaceus*, and *Leuconostoc mesenteroides* subsp. *mesenteroides*; LPLR stands for combination of LPL and *Lactiplantibacillus pentosus*. Microbial enumerations are based on fresh substances. Different lowercase letters in the superscript represent significant differences between different groups (*p* < 0.05). The low, medium, and high concentrations represent 1 × 10^6^, 1 × 10^7^, and 1 × 10^8^ CFU/g FW, respectively.

Water-soluble carbohydrates as the main substrate were continuously consumed by LAB during the ensiling process. After 60 d of corn stover ensiling, all additive groups showed significantly lower WSC content than CK (*p* < 0.05), decreasing by 3.16–11.75%. Among them, the LPLR group with low concentration had the highest WSC content (6.43% DM) with no significant difference compared to the JCK group (6.39% DM). Furthermore, the LPL group at the medium inoculation level maintained higher WSC levels (6.23% DM) compared to the R group (5.86% DM). At low concentrations, the WSC content in the LPLR group was significantly higher than that in the LPL group (*p* < 0.05). As the inoculation concentration of the LPLR group increased, the WSC decreased. This indicated that a high concentration of microbial additives increased the fermentation efficiency to a certain maximum, thereby masking the component advantages observed at low doses.

Additive groups exhibited significantly higher LA and AA contents than CK (*p* < 0.05), while PA and BA were not detected in any treatment. There was no significant difference in LA content among the medium-concentration LPL group, the LPLR group, and the JCK group (*p* > 0.05). Except for high concentration, the LA content in the LPLR group and the LPL group at other concentrations was significantly higher than in the R group (*p* < 0.05), with no significant difference observed between these inoculated groups (*p* > 0.05). The LPLR group (1 × 10^7^ CFU/g FW) showed the highest AA content (2.97% DM). The group represented a significant increase of 33.20% compared to the CK group (2.23% DM) and was significantly higher than the JCK group (*p* < 0.05). At high concentration, the LPL group produced an AA content of 2.83% DM significantly better than the R group (*p* < 0.05), and 12.30% higher than the LPLR group.

The LAB count in the LPLR group (1 × 10^6^ CFU/g FW) reached 8.72 log_10_ cfu/g FW. This value was 6.21% higher than that of the R group at the same concentration (8.21 log_10_ cfu/g FW). At high concentration, the LAB count of the LPL group reached 8.74 log_10_ cfu/g FW, which was 4.42% higher than the highest value in the R group (8.37 log_10_ cfu/g FW). The JCK group exhibited the lowest AB counts (4.04 log_10_ cfu/g FW), with no significant difference observed compared to the medium-concentration LPL and LPLR groups (*p* > 0.05). The LPLR group was significantly better than the R group at all concentrations (*p* < 0.05). These results indicated that the addition of heterofermentative LAB had a stronger inhibitory effect on aerobic microorganisms than the combination of only homofermentative LAB. Notably, after ensiling, the counts of yeast and mold in all LAB-treated groups were reduced to the limit of detection (2.00 log_10_ cfu/g FW). These levels were significantly lower than the CK group (*p* < 0.05), indicating that all the applied LAB inoculants demonstrated greater efficacy in inhibiting fungal contamination.

### Effects of FAE-producing LAB co-fermentation on nutrient composition of corn stover silage

The chemical composition in silage after ensiling of 60 d was showed in Table 3. At low inoculation level, all additive treatments showed a significant improvement in CP preservation. The JCK, R, and LPLR treatments increased CP content by 9.02, 5.44, and 6.69%, respectively, compared to the CK. The JCK exhibited the highest CP content. In contrast, the CP content of LPLR and R did not differ significantly from CK at medium and high concentrations (*p* > 0.05). The AN content was expressed as a percentage of total nitrogen (TN). The AN content in the medium-concentration LPL, low-concentration LPLR, and JCK treatment groups was significantly lower than the CK group (*p* < 0.05), with decreases of 21.5, 22.60, and 27.96%, respectively.

**TABLE 3:** Effects of different inoculants and inoculation levels on chemical composition of corn stover silage after 60 d of ensiling.

Item	Addition amount	Group				
		CK	JCK	R	LPL	LPLR
Moisture (%)	Low	67.72 ± 1.53^d^	66.52 ± 2.23^a^	67.63 ± 0.91^d^	66.92 ± 1.87^b^	67.63 ± 1.27^d^
	Medium			66.85 ± 0.77^b^	67.52 ± 1.21^d^	67.7 ± 1.53^d^
	High			67.07 ± 0.69^c^	67.25 ± 0.49^c^	68.14 ± 2.73^e^
CP (%)	Low	6.43 ± 0.43^c^	7.01 ± 0.83^a^	6.78 ± 0.38^b^	6.57 ± 0.67^bc^	6.86 ± 0.41^b^
	Medium			6.47 ± 0.59^c^	6.79 ± 0.72^b^	6.44 ± 0.57^c^
	High			6.54 ± 0.35^bc^	6.35 ± 0.49^c^	6.21 ± 0.67^c^
NDF (%)	Low	47.58 ± 2.72^a^	46.58 ± 2.33^bc^	45.35 ± 1.89^de^	46.97 ± 2.34^b^	45.86 ± 0.79^d^
	Medium			45.07 ± 2.53^e^	46.43 ± 1.47^bc^	45.36 ± 1.41^e^
	High			46.58 ± 1.43^bc^	46.85 ± 1.93^b^	45.53 ± 1.74^d^
ADF (%)	Low	28.07 ± 1.53^a^	26.74 ± 1.74^bc^	26.76 ± 1.58^bc^	27.03 ± 0.57^b^	26.53 ± 2.03^c^
	Medium			26.54 ± 2.13^c^	27.35 ± 1.96^b^	26.04 ± 0.85^d^
	High			25.89 ± 0.64^bc^	26.98 ± 1.81^b^	26.77 ± 1.72^c^
HC (%)	Low	19.53 ± 1.47^bc^	19.84 ± 1.33^bc^	19.31 ± 1.21^c^	20.03 ± 1.48^b^	18.78 ± 1.62^d^
	Medium			19.19 ± 2.25^c^	19.85 ± 1.71^bc^	19.33 ± 1.27^c^
	High			20.48 ± 0.79^a^	19.71 ± 2.47^bc^	18.82 ± 1.53^d^
AN (%)	Low	0.93 ± 0.17^a^	0.67 ± 0.23^d^	0.87 ± 0.15^ab^	0.85 ± 0.11^ab^	0.74 ± 0.07^d^
	Medium			0.84 ± 0.07^ab^	0.72 ± 0.14^d^	0.79 ± 0.13^bc^
	High			0.92 ± 0.17^a^	0.81 ± 0.15^bc^	0.85 ± 0.21^ab^
Ash (%)	Low	3.23 ± 0.45^a^	3.25 ± 0.18^a^	3.37 ± 0.68^a^	3.33 ± 0.17^a^	3.17 ± 0.21^a^
	Medium			3.31 ± 0.44^a^	3.15 ± 0.28^a^	3.23 ± 0.18^a^
	High			3.19 ± 0.53^a^	3.34 ± 0.13^a^	3.31 ± 0.09^a^

CP, crude protein; NDF, neutral detergent fiber; ADF, acid detergent fiber; HC, hemicellulose; AN, ammonia nitrogen; JCK stands for commercial microbial agent control group; R stands for *Lactiplantibacillus pentosus* alone; LPL stands for combination of *Lactiplantibacillus plantarum*, *Pediococcus pentosaceus*, and *Leuconostoc mesenteroides* subsp. *mesenteroides*; LPLR stands for combination of LPL and *Lactiplantibacillus pentosus*. Different lowercase letters in the superscript represent significant differences between different groups (*p* < 0.05). The low, medium, and high concentrations represent 1 × 10^6^, 1 × 10^7^, and 1 × 10^8^ CFU/g FW, respectively.

The NDF content of the R group and the LPLR group was significantly lower than other groups at all concentrations (*p* < 0.05). Except at low concentration, there was no significant difference in NDF content between the R and LPLR groups (*p* > 0.05). The ADF content in all additive treatment groups was significantly lower than CK (*p* < 0.05). The LPLR group exhibited the lowest ADF content at the medium concentration (26.04%), which was significantly lower than that of the other groups (*p* < 0.05). There was no significant difference between the R group (except the medium concentration) and the LPLR group (*p* > 0.05). Except at the medium concentration, the LPLR groups showed significant differences compared to the other groups (*p* < 0.05). The fiber content in JCK was higher than that in groups supplemented with FAE-producing lactobacillus. In addition, there were no significant differences in ash content among the treatment groups (*p* > 0.05).

### Effects of FAE-producing LAB on aerobic stability of corn stover silage

Supplementary Figure 2 showed the temperature changes of each treatment group during 7 d of aerobic exposure. The LPLR and LPL groups at medium concentration showed no significant temperature changes during 7 d of aerobic exposure. The CK group lost aerobic stability the fastest among all groups.

At the aerobic exposure period, the pH of all groups exhibited an increasing trend. The LPL and LPLR maintained low pH levels at the medium inoculation amount (Table 4), which were significantly lower than those of the other groups (*p* < 0.05). At 7 d, the LPLR group had the highest LA content (8.32%) at the medium inoculation level, which was significantly higher than CK (*p* < 0.05). The AN/TN value of each group increased during the aerobic exposure period. At 7 d of aerobic exposure, the LPL group showed the lowest AN/TN value (0.97%) at the medium inoculation level.

**TABLE 4:** Effects of different inoculants and inoculation levels on aerobic stability of corn stover silage.

Group	Addition amount	Parameter					
		pH	LA (%)	AN (% TN)	LAB (log_10_ cfu/g FW)	AB (log_10_ cfu/g FW)	Yeast (log_10_ cfu/g FW)
Aerobic exposure for 3 d							
CK		4.07 ± 0.13^a^	7.58 ± 0.46^e^	1.27 ± 0.34^a^	8.43 ± 0.57^c^	6.07 ± 0.58^a^	3.45 ± 0.53^b^
JCK		3.91 ± 0.21^b^	8.03 ± 0.57^d^	1.05 ± 0.13^b^	7.85 ± 0.83^e^	5.77 ± 0.67^b^	3.07 ± 0.27^d^
R	Low	3.94 ± 0.11^Ab^	8.23 ± 0.42^Bd^	1.07 ± 0.15^Bb^	8.03 ± 0.35^Ce^	5.76 ± 0.55^Ab^	3.28 ± 0.63^Bc^
	Medium	3.85 ± 0.17^Bbc^	8.59 ± 0.49^Ac^	1.13 ± 0.27^Aab^	8.74 ± 0.58^Aa^	5.48 ± 0.71^Bc^	3.57 ± 0.26^Aa^
	High	3.98 ± 0.09^Ab^	8.25 ± 0.47^Bd^	0.98 ± 0.17^Cbc^	8.57 ± 0.29^Bb^	5.79 ± 0.68^Ab^	3.08 ± 0.77^Cd^
LPL	Low	3.85 ± 0.12^Bbc^	8.07 ± 0.85^Cd^	0.96 ± 0.11^Bbc^	8.66 ± 0.49^Aab^	5.75 ± 0.59^Ab^	2.63 ± 0.58^Be^
	Medium	3.75 ± 0.07^Cc^	8.94 ± 0.59^Aa^	0.81 ± 0.14^Cd^	8.72 ± 0.53^Aa^	5.07 ± 0.79^Be^	2.32 ± 0.45^Cf^
	High	3.92 ± 0.14^Abc^	8.73 ± 0.58^Bb^	1.05 ± 0.15^Ab^	7.96 ± 0.65^Be^	5.83 ± 1.23^Ab^	2.94 ± 0.68^Ad^
LPLR	Low	3.72 ± 0.21^Cc^	7.65 ± 1.23^Ce^	0.93 ± 0.07^Bc^	8.3 ± 0.68^Bd^	5.45 ± 0.72^Bc^	2.51 ± 0.61^Be^
	Medium	3.81 ± 0.05^Bc^	8.82 ± 0.56^Aab^	0.88 ± 0.13^Bc^	8.65 ± 0.39^Aab^	5.29 ± 1.32^Cd^	2.18 ± 0.39^Cf^
	High	3.87 ± 0.13^Abc^	8.21 ± 0.67^Bd^	1.18 ± 0.21^Aab^	8.23 ± 1.01^Bd^	5.78 ± 0.78^Ab^	3.03 ± 0.45^Ad^
Aerobic exposure for 7 d							
CK		4.38 ± 0.23^a^	5.88 ± 0.49^e^	1.53 ± 0.34^a^	12.33 ± 1.37^a^	8.07 ± 0.58^a^	6.27 ± 0.63^a^
JCK		4.04 ± 0.07^bc^	7.65 ± 0.93^c^	1.23 ± 0.31^bc^	9.43 ± 0.57^d^	7.84 ± 0.38^c^	5.85 ± 0.71^c^
R	Low	4.04 ± 0.13^Bb^	7.73 ± 0.42^Bc^	1.37 ± 0.15^Ab^	9.65 ± 0.35^Ad^	7.65 ± 0.55^Bc^	5.78 ± 0.49^Bc^
	Medium	3.96 ± 0.17^Cc^	8.09 ± 0.49^Ab^	1.13 ± 0.19^Bc^	9.17 ± 0.58^Ce^	6.84 ± 0.77^Cf^	5.57 ± 0.26^Cd^
	High	4.15 ± 0.17^Ab^	7.82 ± 0.37^Bc^	1.36 ± 0.27^Ab^	9.44 ± 0.39^Bd^	7.79 ± 0.58^Ac^	6.08 ± 0.77^Ab^
LPL	Low	3.92 ± 0.12^Ac^	8.07 ± 0.85^Bb^	1.21 ± 0.11^Abc^	10.66 ± 0.49^Ab^	7.65 ± 0.79^Ad^	5.73 ± 0.58^Acd^
	Medium	3.83 ± 0.07^Bd^	8.24 ± 0.59^Aa^	0.97 ± 0.14^Cd^	9.98 ± 0.53^Bc^	6.89 ± 0.79^Cf^	5.42 ± 0.48^Be^
	High	3.92 ± 0.14^Ac^	7.73 ± 0.58^Cc^	1.12 ± 0.15^Bc^	9.73 ± 0.45^Cd^	7.43 ± 1.23^Bd^	5.84 ± 0.68^Ac^
LPLR	Low	3.89 ± 0.21^Bc^	7.85 ± 1.23^Bc^	1.08 ± 0.07^Bc^	8.77 ± 0.68^Cd^	7.81 ± 0.72^Ab^	5.51 ± 0.61^Bd^
	Medium	3.81 ± 0.15^Cd^	8.32 ± 0.56^Aa^	1.04 ± 0.13^Bc^	10.45 ± 1.33^Ab^	7.25 ± 1.32^Be^	5.49 ± 0.39^Bde^
	High	4.07 ± 0.03^Ab^	7.34 ± 0.67^Cd^	1.28 ± 0.27^Abc^	9.27 ± 1.01^Be^	7.86 ± 0.78^Ab^	6.03 ± 0.45^Ab^

FW, fresh weight; LA, lactic acid; AN, ammonia nitrogen; LAB, lactic acid bacteria; AB, aerobic bacteria; JCK stands for commercial microbial agent control group; R stands for *Lactiplantibacillus pentosus* alone; LPL stands for combination of *Lactiplantibacillus plantarum*, *Pediococcus pentosaceus*, and *Leuconostoc mesenteroides* subsp. *mesenteroides*; LPLR stands for combination of LPL and *Lactiplantibacillus pentosus*. Different lowercase letters in the superscript represent significant differences between different groups (*p* < 0.05). Intra-group differences are indicated by capital letters. The low, medium, and high concentrations represent 1 × 10^6^, 1 × 10^7^, and 1 × 10^8^ CFU/g FW, respectively. Mold count is not presented, as they were uniformly low and not significantly different (*p* > 0.05) in all treatments after aerobic exposure.

After 60 d of ensiling, the counts of yeast and AB decreased to a lower level. At the aerobic exposure period, the counts of LAB in each treatment group increased to varying degrees. Among them, the CK group showed the most significant increase. The counts of AB and yeast also continued to increase during the aerobic stage in the CK group, which was significantly higher than the other groups *(p* < 0.05). At 7 d of aerobic exposure, the LPL group had the lowest amount of AB and yeast at the medium inoculation level. The counts of AB and yeast in the LPLR and LPL groups were significantly lower than those the JCK group at all concentrations (*p* < 0.05).

### Comprehensive evaluation of FAE-producing LAB fermented corn stover silage

Comprehensive evaluation was determined and ranked using a membership function (Table 5). The selection and weighting of variables in this evaluation were based on the PCA (Supplementary Figure 1). The CK group showed the lowest score (0.302) among all 11 treatments. In contrast, the LPLR treatment at medium inoculation level achieved the highest score (0.696). Scores for the other concentrations of LPLR were 0.587 and 0.339, ranking fifth and eighth, respectively. Although the JCK group demonstrated efficacy during the fermentation phase, it exhibited poor aerobic stability at aerobic exposure, resulting in a second-place ranking. The LPL group at medium inoculation level ranked third. Therefore, LPLR at 1 × 10^7^ CFU/g FW inoculation amount was identified as the optimal formula, comprising the homolactic bacteria *L. plantarum* and *P. pentosaceus*, the heterolactic bacteria *Leuc. mesenteroides*, and the FAE-producing *L. pentosus*.

**TABLE 5:** Comprehensive evaluation of corn stover silage quality under different inoculants and inoculation levels.

Group	Addition amount	Ensiling for 60 d												Aerobic exposure for 7 d						AVG	Ranking
		WSC	CP	DM	LA	AA	ADF	NDF	pH	HC	AN	Ash	PA	AB	LA	LAB	AN	pH	Yeast		
CK		1	0.275	0.259	0	0.253	0	0	0	0.559	0	0.636	-	0	0	1	0	0	0	0.302	11
JCK		0.679	1	1	1	0.273	0.610	0.398	0.903	0.376	1	0.545	-	0.187	0.725	0.185	1	0.596	0.494	0.690	2
R	Low	0.269	0.713	0.315	0.401	0	0.601	0.888	0.387	0.688	0.231	0.00	-	0.341	0.758	0.247	0.231	0.596	0.576	0.314	10
	Medium	0	0.325	0.796	0.474	0.343	0.702	1	0.548	0.759	0.346	0.273	-	1	0.906	0.112	0.346	0.737	0.824	0.485	7
	High	0.128	0.413	0.660	0.579	0.242	1	0.398	0.194	0	0.038	0.818	-	0.228	0.795	0.188	0.038	0.404	0.224	0.326	9
LPL	Low	0.423	0.450	0.753	0.691	0.192	0.477	0.243	0.710	0.265	0.308	0.182	-	0.341	0.898	0.531	0.308	0.807	0.635	0.577	6
	Medium	0.474	0.725	0.383	0.961	0.606	0.330	0.458	1	0.371	0.808	1	-	0.959	0.967	0.340	0.808	0.965	1	0.686	3
	High	0.231	0.175	0.549	0.895	0.859	0.500	0.291	0.839	0.453	0.462	0.136	-	0.520	0.758	0.270	0.462	0.807	0.506	0.657	4
LPLR	Low	0.731	0.813	0.315	0.566	0.354	0.706	0.685	0.677	1	0.731	0.909	-	0.211	0.807	0	0.731	0.860	0.894	0.587	5
	Medium	0.372	0.288	0.272	0.928	1	0.931	0.884	0.839	0.676	0.538	0.636	-	0.667	1	0.472	0.538	1	0.918	0.696	1
	High	0.090	0	0	0.329	0.545	0.596	0.817	0.516	0.976	0.308	0.273	-	0.171	0.598	0.140	0.308	0.544	0.282	0.339	8

WSC, water soluble Carbohydrate; CP, crude protein; DM, dry matter; LA, lactic acid; AA, acetic acid; ADF, acid detergent fiber; NDF, neutral detergent fiber; HC, hemicellulose; AN, ammonia nitrogen; PA, propanoic acid; AB, aerobic bacteria; LAB, lactic acid bacteria; AVG stands for membership average. JCK stands for commercial microbial agent control group; R stands for *Lactiplantibacillus pentosus* alone; LPL stands for combination of *Lactiplantibacillus plantarum*, *Pediococcus pentosaceus*, and *Leuconostoc mesenteroides* subsp. *mesenteroides*; LPLR stands for combination of LPL and *Lactiplantibacillus pentosus*.

**FIGURE 3:**
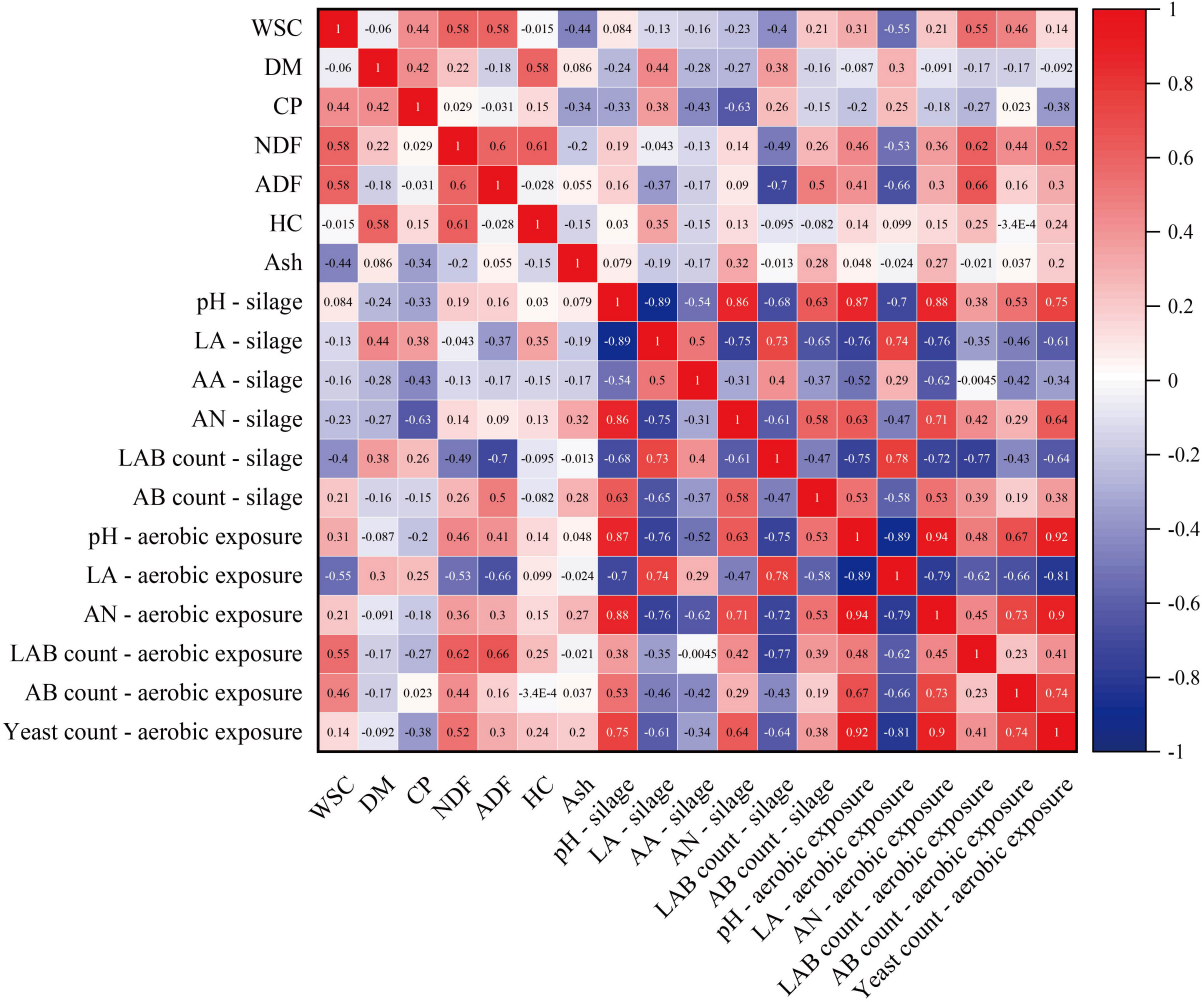
Pearson correlation analysis of fermentation quality, nutritional composition, and aerobic stability in corn stover silage. The red squares represent positive correlations (0 < *r* < 1), while the blue squares represent negative correlations (-1 < *r* < 0). WSC, water soluble carbohydrate; CP, crude protein; DM, dry matter; LA, lactic acid; AA, acetic acid; ADF, acid detergent fiber; NDF, neutral detergent fiber; HC, hemicellulose; AN, ammonia nitrogen; PA, propanoic acid; AB, aerobic bacteria; LAB, lactic acid bacteria.

Pearson correlation analysis (Figure 3) demonstrated complex and significant correlations among the nutritional components, fermentation quality, and aerobic stability during ensiling. After 60 d of ensiling, pH exhibited a highly significant negative correlation with LA content (*r* = -0.89, *p* < 0.001) and a strong significant positive correlation with AN content (*r* = 0.86, *p* < 0.001). Additionally, a significant negative correlation was observed between ADF content and LAB counts (*r* = -0.70, *p <* 0.05). After 7 d of aerobic exposure, pH showed a strong positive correlation with yeast count (*r* = 0.92, *p* < 0.001) and a strong negative correlation with LA content (*r* = -0.89, *p* < 0.001). A strong negative correlation was observed between LA concentration and yeast count (*r* = -0.81, *p* < 0.01). The AN content was positively correlated with yeast counts (*r* = 0.90, *p* < 0.001). Furthermore, the nutritional composition and fermentation quality indicators of silage at 60 d were correlated with the indicators after 7 d of aerobic exposure. The pH after 60 d of silage fermentation was positively correlated with yeast counts (*r* = 0.75, *p* < 0.01) after 7 d of exposure. Higher LA accumulation was associated with the pH rise during the aerobic exposure period (*r* = -0.76, *p* < 0.01) and the maintenance of residual LA (*r* = 0.74, *p* < 0.01). The counts of LAB after 60 d of ensiling showed a protective effect with LA maintenance during the aerobic exposure period (*r* = 0.78, *p* < 0.01) and negative correlations with yeast proliferation (*r* = -0.64, *p* < 0.05). Moreover, AN accumulation during the fermentation phase showed a significant positive correlation with both AN content (*r* = 0.71, *p* < 0.05) and yeast counts (*r* = 0.64, *p* < 0.05) at the exposure phase.

In summary, Pearson correlation analysis highlighted the complex interaction network between the nutritional composition of silage feed (especially fiber components and protein degradation products), fermentation quality (such as pH, LA, and LAB), and their sensitivity to aerobic spoilage.

## Discussion

Five LAB strains were isolated: four homofermentative and one heterofermentative. In this study, *Lactiplantibacillus pentosus* AR1 was isolated by plate screening. It was reported that high-producing feruloyl esterase microbes were mainly known to be fungi, such as *Aspergillus terreus* (70 mU/mL) and *Aspergillus niger* (28 mU/mL) (Pérez-Rodríguez et al., 2016). However, the FAE-producing bacterial strains are limited, and their enzymatic activity is generally low. The FAE activity of strain AR1 (7.11 mU/mL) is significantly higher than that of previously reported bacteria *Pediococcus acidilactici* (4.32 mU/mL) and comparable to the activity levels of *Lactiplantibacillus plantarum* (8.34 mU/mL) and *Lactobacillus brevis* (7.56 mU/mL) (Ding et al., 2019). These results indicate that AR1 is a LAB resource with high potential for efficient feruloyl esterase production, providing a new candidate strain for the development of bacteria-based fiber-degrading additives.

Most recent studies on co-fermentation with FAE-producing bacteria have mainly involved combinations of FAE-producing heterofermentative with non-FAE-producing strains (Jin et al., 2015), or mixtures of FAE-producing homofermentative and heterofermentative strains (Ding et al., 2019). However, there is no report on the synergy effects between FAE-producing lactobacillus (homofermentative) and other LAB during silage. In this study, the synergistic effect of FAE-producing lactobacillus and different types of LAB improved silage fermentation quality, reduce the lignocellulose content of corn stover silage, and enhance aerobic stability.

The addition of LAB alone or in combination can effectively reduce pH of silage (Okoye et al., 2023a). In addition, the added LAB can produce large amounts of organic acids and other antibacterial products to inhibit protease activity and the growth of harmful microorganisms, thereby improving the quality of silage feed (Dunière et al., 2013). In this study, the pH of each treatment group at different inoculation amounts was lower than that in the CK group. This may be because each additive group contains the homofermentative LAB. The strains efficiently convert soluble carbohydrates in corn stover (such as glucose, xylose, cellobiose, and arabinose) into LA by the Embden-Meyerhof-Parnas pathway, thereby rapidly lowering the pH at the early stages of fermentation (Guo et al., 2022). Meanwhile, heterofermentative LAB also utilize sugars to produce substances such as AA, 1,2-propylene glycol, and ethanol, which effectively inhibit the growth of molds and other aerobic microorganisms (Kung et al., 2018). Previous studies have reported that *Leuc. mesenteroides* produce large amounts of extracellular polysaccharides such as glucan (Li et al., 2020). These polymers are hard to volatilize during ashing and can remain as carbonates, raising the ash content. This may be the reason for the significant difference in ash content between the LM and LPA groups.

The NDF and ADF content in feed can accurately reflect the fiber composition in feed and are key parameters for evaluating its nutritional value. Some researchers have suggested that LAB can modify the fiber structure of stover to reduce fiber content by anaerobic fermentation (Muck et al., 2018). This is consistent with this experiment. Previous studies have mostly focused on using various organic acid and cellulases in combination with LAB for silage to reduce the fiber content of the substrate (Zi et al., 2021; Li Y. et al., 2022). However, the use of enzymes and organic reagents is often limited by high costs and sensitivity to reaction conditions in industrial applications (Wei et al., 2026). In this study, adding the selected FAE-producing *Lactiplantibacillus* alone or in combination significantly reduced lignocellulose content, which is more cost-effective and favorable for industrial production compared to enzymatic degradation methods. Li et al. (2019) found that the addition of FAE-producing LAB to corn stover silage reduced the NDF content, indicating a promoting effect on cellulose degradation. This finding is consistent with the results of the present study.

High CP along with low AN levels are associated with greater nutritional value in feed (Jin et al., 2024). In this study, except for the JCK group, the R and LPLR groups with low inoculation concentrations, and the LPL group with medium addition, where CP was significantly higher than CK (*p* < 0.05), the CP content in other addition concentration groups showed no significant difference compared with CK. However, adding functional LAB significantly reduced the AN content, which is consistent with the findings of Tahir et al. (2025). This indicates that the LAB additive group can effectively inhibit protease activity in the native aerobic microorganisms, thereby reducing protein degradation in the feed.

After 7 d of aerobic exposure, the yeast count showed a significant positive correlation with both AN content and pH value. This correlation may be due to the reason that yeast can use LA as a carbon source for growth and metabolism. In this study, as LA content decreased, the pH value increased. This elevation in pH disrupts the inhibitory role of the originally acidic environment. Consequently, aerobic microorganisms proliferate rapidly and secrete proteases to degrade CP in the silage, resulting in an increased AN content. Therefore, aerobic stability during silage is associated with the growth of yeast. However, different LAB additives have significant differences in aerobic stability.

In this study, the JCK group showed a more significant effect during the ensiling stage, with better comprehensive value than other groups. However, its stability during the aerobic exposure period was poor, significantly worse than the treatment group with heterolactic bacteria added. The JCK group contains *Enterococcus faecalis*, which has the ability to produce AA. However, as a facultative heterofermentative LAB, its efficiency in AA production is lower than that of the obligate heterofermentative R3 (Muck et al., 2018). Furthermore, the JCK group contains *Candida utilis*. During aerobic exposure, yeasts utilized lactic acid for growth and reproduction (Cheirsilp et al., 2003), leading to a proliferation in the yeast population. These factors explain the poor aerobic stability in the JCK group.

In this study, the FAE-producing *Lactiplantibacillus pentosus* AR1 had a significant synergistic effect with other homofermentative and heterofermentative LAB during the silage fermentation system. At the early stage of silage, *L. pentosus* AR1 synergistically with other homofermentative LAB utilized sugars to produce pyruvate through glycolysis. Pyruvate was then reduced to lactic acid through lactic acid fermentation, thereby promoting bacterial growth and lowering the pH. Lower pH inhibited the proliferation of undesirable microorganisms and ensured silage quality. At the middle and late stages of silage, AR1 strain secreted the ferulic acid esterase to degrade lignocellulose. The enzyme specifically hydrolyzed the ester bonds between ferulic acid and hemicellulose (such as arabinoxylan) in the plant cell wall (Oliveira et al., 2019). The hydrolyzation process disrupted the cross-linked structure of lignin-carbohydrate complexes and released more fermentable sugars to provide sufficient substrates for the continuous acid production by both homofermentative and heterofermentative LAB. The synergistic effect among these strains explains why the AA content in the LPLR group is significantly higher than in the other treatment groups.

Except for the high AA content, the LPLR group further enhanced its aerobic stability by producing abundant antioxidants (ferulic acid). Ferulic acid inhibits the growth of other bacteria by disrupting cell permeability and interfering with protein synthesis. At the early stage, it temporarily inhibited other LAB, affecting the beginning of lactic acid synthesis. This explains the lower lactic acid content in the LPLR group compared to the LPL group. During aerobic exposure, this phenolic acid inhibited molds, yeasts, and AB to improve aerobic stability, which is consistent with Li et al. (2021).

In the large-scale production of silage, the inoculation concentration is one of the key indicators that determine cost-effectiveness. A lower inoculation concentration of exogenous lactobacillus addition makes it difficult to quickly establish an advantage in the complex native microbial community, leading to poor fermentation. On the other hand, higher inoculation concentration can lead to a significant increase in production costs. For these reasons, it is important to determine the optimal amount of microbial agent for application. Therefore, we designed different LAB dosages to select the optimal concentration for industrial production. In the present study, this effect of inoculation concentration was observed in the LPLR treatments.

For LPLR group, homofermentative LAB were the dominant species. Thus, high amount addition of LPLR could help rapidly establish a competitive advantage of exogenous homofermentative LAB at the early stage of silage (Mu et al., 2020). Thus, lactic acid was rapidly produced to lower pH, which effectively inhibited the growth and AA production of epiphytic heterofermentative bacteria, such as *Weissella* (Okoye et al., 2023b). Therefore, the AA content in the high inoculation amount was significantly lower than that in the medium and low concentration groups.

During the aerobic exposure period, the low concentration of AA could not effectively inhibit aerobic microorganisms (Zhang et al., 2021). Furthermore, the high inoculation concentration led to excessive consumption of fermentation substrates at the early stage. Due to the substrate limitations, LAB grew with difficulty under the aerobic condition. Meanwhile, yeasts and aerobic microorganisms utilized the LA to proliferate rapidly, leading to an increase in pH.

The synergistic effect of these microbial activities resulted in high-quality silage with good fermentation characteristics, preserved nutrients, low cellulose content, and excellent aerobic stability. These findings may be extrapolated to agricultural wastes similar to corn stover, which contain low levels of soluble carbohydrates and high amounts of lignocellulose, such as wheat straw, rice straw, and reed straw (Liu et al., 2024). Further research could optimize the enzyme production conditions and develop more efficient functional LAB for silage to provide guidance for practical production applications.

## Conclusion

In summary, FAE-producing LAB addition provides positive effects on co-fermentation of homofermentative and heterofermentative LAB. The formula combination of *Lactiplantibacillus plantarum, Pediococcus pentosaceus, Leuconostoc mesenteroides* subsp. *mesenteroides and Lactiplantibacillus pentosus* (1 × 10^7^ CFU/g FW, 1:1:1:1 ratio) exhibited a significant improvement on silage compared to the commercial bacterial agents. These findings demonstrate that optimizing strain combinations not only enhances fermentation quality but also increases nutrient content, providing a scientific guidance for high-quality silage production.

## Data Availability

The datasets presented in this study can be found in online repositories. The names of the repository/repositories and accession number(s) can be found at: https://www.ddbj.nig.ac.jp/, LC909648-LC909652.
