# Supplementary material for: Feruloyl esterase-producing lactobacillus screening and its synergistic effect with homolactic and heterolactic bacteria on corn stover silage
Source: Front Microbiol. 2026 Mar 6;17:1755745. doi: 10.3389/fmicb.2026.1755745 (PMC13002831; doi:10.3389/fmicb.2026.1755745)
Supplement: Supplementary file 6 [file Data_Sheet_6.pdf]

Feruloyl esterase-producing lactobacillus screening and its synergistic effect with homolactic and heterolactic bacteria on corn stover silage

Wang Lin[1], Cai Zilu[2], Li Fusheng[2], Ke Zhuang[1], Tao Ran[3], Ye Bin[4], Yuan Cansheng[1,*], He Qin[2,*]

College of Rural Revitalization, Jiangsu Open University, Nanjing, Jiangsu 210036, PR China[1], Sanya Institute of Nanjing Agricultural University, Department of Microbiology, Key Lab of Microbiological Engineering of Agricultural Environment, Ministry of Agriculture, College of Life Sciences, Nanjing Agricultural University, Nanjing 210095, PR China[2], Institute of Chemical Industry of Forest Products, CAF, Nanjing 210042, PR China[3], Institute of Microbe and Host Health, Linyi University, Linyi, Shandong 276005, PR China[4]

Table S1. Characteristics of silage before and after fermentation

Item	0 d	Post-fermentation (60 d)					
	Substrate	CK	LPA	LPP	LM	LAL	LPL
pH	6.47±0.05	3.72±0.07	3.68±0.02	3.67±0.13	3.77±0.15	3.62±0.11	3.56±0.08
Moisture (%)	63.47±0.68	65.61±0.51^{b}	64.43±0.74^{a}	63.52±0.67^{a}	64.15±0.74^{a}	63.77±0.38^{a}	63.33±1.13^{a}
CP (%)	6.16±0.43	7.31±0.95	7.24±0.28	7.47±0.62	7.23±0.57	7.36±0.48	7.39±0.57
WSC (%)	13.87±0.83	7.52±0.36^{a}	6.93±0.47^{b}	7.07±0.85^{b}	6.74±0.37^{b}	7.09±0.73^{b}	7.15±0.54^{b}
NDF (%)	49.34±0.58	50.31±2.81^{a}	48.07±4.44^{bc}	48.23±2.77^{bc}	49.54±1.37^{ab}	49.23±1.33^{ab}	47.94±1.58^{c}
ADF (%)	29.73±0.59	26.85±1.69^{a}	26.02±2.07^{b}	25.61±1.47^{bc}	26.87±1.43^{a}	25.37±2.53^{c}	25.48±1.69^{c}
HC (%)	19.88±0.58	23.56±1.12^{a}	22.05±2.37^{c}	22.62±1.30^{b}	22.69±0.84^{b}	23.86±2.20^{a}	22.46±1.63^{bc}
Ash (%)	0.92±0.31	3.82±0.52^{ab}	3.74±0.73^{b}	3.77±0.46^{ab}	3.85±0.67^{a}	3.78±0.31^{ab}	3.81±0.57^{ab}
LAB (log_{10} cfu/g FW)	5.23±0.78	7.93±0.41^{c}	9.08±0.69^{a}	8.84±0.65^{ab}	8.58±0.79^{b}	9.23±0.47^{a}	9.18±0.82^{a}
AB (log_{10} cfu /g FW)	6.96±0.87	4.73±0.25^{a}	4.45±0.68^{b}	4.34±0.61^{bc}	4.77±0.87^{a}	4.23±0.35^{bc}	4.11±0.48^{c}
Yeast (log_{10} cfu /g FW)	4.53±0.74	<2.00	<2.00	<2.00	<2.00	<2.00	<2.00
Molds (log_{10} cfu /g FW)	6.75±0.79	<2.00	<2.00	<2.00	<2.00	<2.00	<2.00

FW, fresh weight; WSC, water soluble carbohydrate; CP, crude protein; ADF, acid detergent fiber; NDF, neutral detergent fiber; HC, hemicellulose; AN, ammonia nitrogen; PA, propanoic acid; AB, aerobic bacteria; LAB, lactic acid bacteria. Substrate, whole corn stover before

silage. *Lactiplantibacillus plantarum*, *Pediococcus pentosaceus*, *Pediococcus acidilactici*, and *Leuconostoc mesenteroides* subsp. *mesenteroides* are abbreviated as LP, PP, PA, and LM, respectively. LPA, LPP, LAL, and LPL in the conclusion and analysis section below are the abbreviations of the above additive groups, corresponding to (LP+PA), (LP+PP), (LP+PA+LM), and (LP+PP+LM), respectively.

Table S2. Effects of different inoculants on aerobic stability of corn stover silage

Item	Group					
	CK	LPA	LPP	LM	LAL	LPL
pH	4.58 ± 0.08^{a}	4.30 ± 0.07^{c}	4.24 ± 0.09^{cd}	4.45 ± 0.07^{b}	4.17 ± 0.06^{d}	4.15 ± 0.08^{d}
LA (%)	2.27 ± 0.24^{d}	3.48 ± 0.13^{b}	3.68 ± 0.23^{b}	3.10 ± 0.10^{c}	3.10 ± 0.10^{c}	4.55 ± 0.19^{a}
AN (%)	11.86 ± 0.31^{a}	10.09 ± 0.38^{c}	9.35 ± 0.08^{d}	10.52 ± 0.19^{b}	9.23 ± 0.10^{d}	8.59 ± 0.13^{e}
LAB ($\log_{10}$ cfu /g FW)	9.89 ± 0.68^{ab}	6.32 ± 0.21^{d}	7.29 ± 0.23^{c}	8.10 ± 0.40^{c}	9.24 ± 0.82^{b}	10.19 ± 0.37^{a}
Yeast ($\log_{10}$ cfu /g FW)	7.44 ± 0.42^{b}	7.92 ± 0.20a	8.01 ± 0.29^{a}	5.86 ± 0.09^{c}	5.00 ± 0.14^{d}	4.45 ± 0.38^{e}
AB ($\log_{10}$ cfu /g FW)	4.58 ± 0.08^{a}	10.62 ± 0.54^{a}	10.53 ± 0.18^{a}	9.63 ± 0.15^{bc}	9.83 ± 0.41^{b}	9.04 ± 0.11^{c}

FW, fresh weight; LA, lactic acid; AN, ammonia nitrogen; LAB, lactic acid bacteria; AB, aerobic bacteria. *Lactiplantibacillus plantarum*, *Pediococcus pentosaceus*, *Pediococcus acidilactici*, and *Leuconostoc mesenteroides* subsp. *mesenteroides* are abbreviated as LP, PP, PA, and LM, respectively. LPA, LPP, LAL, and LPL in the conclusion and analysis section below are the abbreviations of the above additive groups, corresponding to (LP+PA), (LP+PP), (LP+PA+LM), and (LP+PP+LM), respectively.

Table S3. Comprehensive evaluation of corn stover silage quality

Group	Addition Amount	Ensiling for 60 d											Aerobic exposure for 15 d						AVG	ranking
		WSC	CP	DM	LA	AA	ADF	NDF	pH	HC	AN	Ash	AB	LA	LAB	AN	pH	Yeast		
CK	Low	1	0.21	0	0.44	0.32	0	0.61	0.48	0	0.31	1	0	0	0.44	0.37	0	0.48	0.34	6
LPA	Low	0.48	0.52	0.71	0.57	0	0.24	0.48	0.59	1	0	0.78	0.42	0.58	0.65	0.21	0.48	0	0.48	4
LPP	Low	0	0.57	0.63	1	0.36	0.39	0.52	0.64	0.41	0.43	0.85	0.56	0.64	1	0.64	0.64	0.52	0.57	3
LM	Low	0.32	0	0.47	0	1	0.42	0	0	0.34	0.47	0.64	0.64	0.24	0	0.44	0.5	1	0.36	5
LAL	Low	0.58	0.61	0.56	0.63	0.74	0.64	1	1	0.72	1	0.71	0.48	0.78	0.54	0	1	0.78	0.69	2
LPL	Low	0.52	1	1	0.68	0.64	1	0.76	0.72	0.63	0.47	0	1	1	0.62	1	0.72	0.64	0.72	1

WSC, water soluble Carbohydrate; CP, crude protein; DM, dry matter; LA, lactic acid; AA, acetic acid; ADF, acid detergent fiber; NDF, neutral detergent fiber; HC, hemicellulose; AN, ammonia nitrogen; AB, aerobic bacteria; LAB, lactic acid bacteria; AVG stands for membership average. The low addition amount represents 1×10^6 CFU/g FW. *Lactiplantibacillus plantarum*, *Pediococcus pentosaceus*, *Pediococcus acidilactici*, and *Leuconostoc mesenteroides* subsp. *mesenteroides* are abbreviated as LP, PP, PA, and LM, respectively. LPA, LPP, LAL, and LPL in the conclusion and analysis section below are the abbreviations of the above additive groups, corresponding to (LP+PA), (LP+PP), (LP+PA+LM), and (LP+PP+LM), respectively.

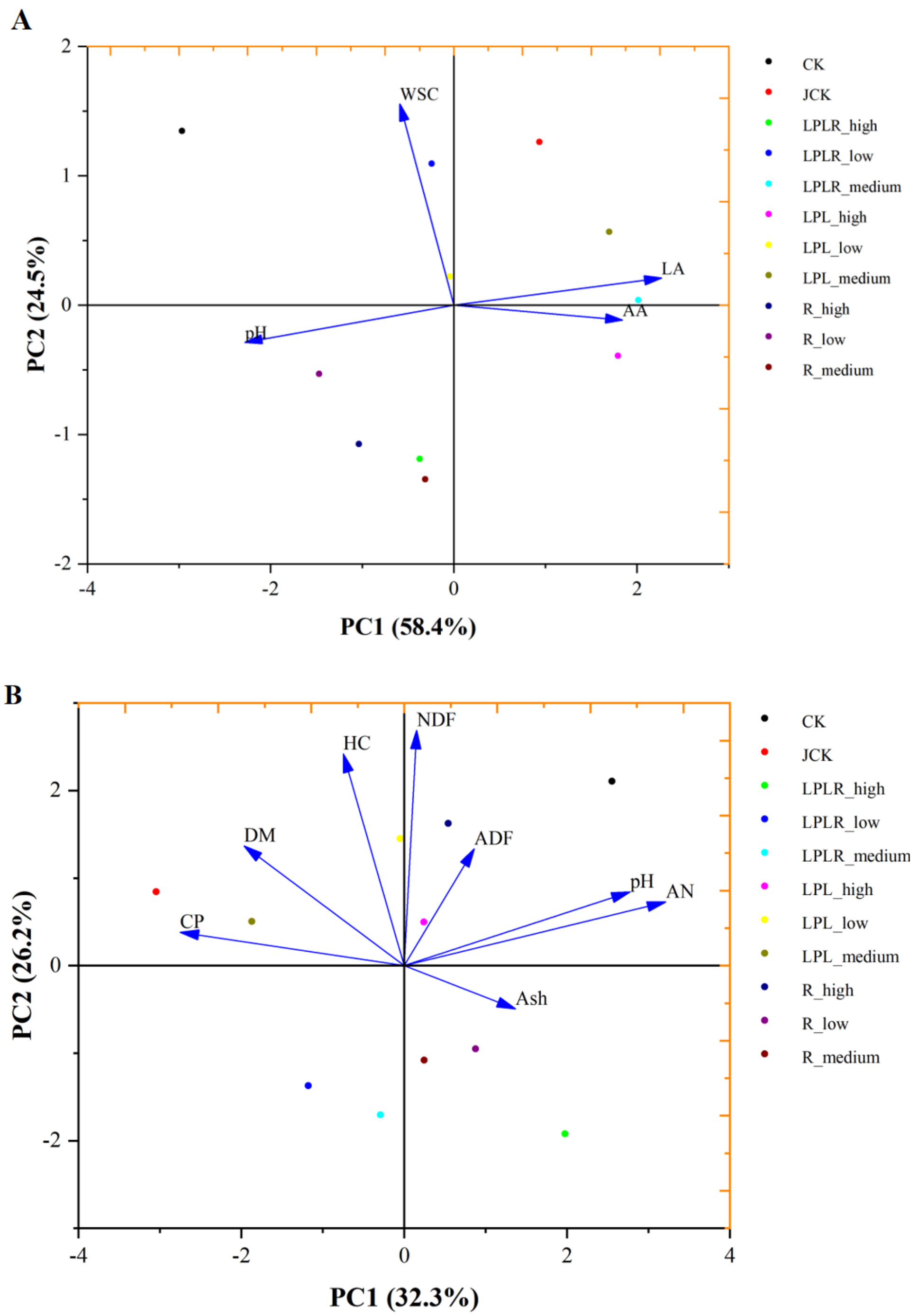

A
WSC
LA
AA
pH
PC1 (58.4%)
PC2 (24.5%)
CK
JCK
LPLR_high
LPLR_low
LPLR_medium
LPL_high
LPL_low
LPL_medium
R_high
R_low
R_medium
B
NDF
HC
DM
ADF
pH
AN
CP
Ash
PC1 (32.3%)
PC2 (26.2%)
CK
JCK
LPLR_high
LPLR_low
LPLR_medium
LPL_high
LPL_low
LPL_medium
R_high
R_low
R_medium

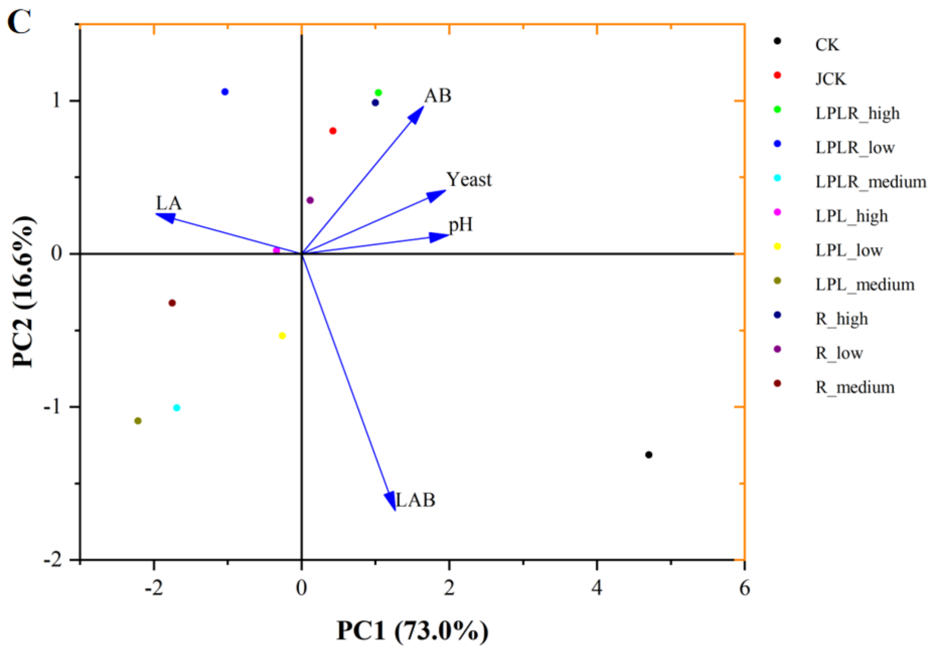

Figure S1. Principal Component Analysis

(A) silage fermentation characteristics after 60 d; (B) nutritional quality of silage after 60 d; (C) aerobic stability of silage after 60 d; WSC, water soluble carbohydrate; CP, crude protein; DM, dry matter; LA, lactic acid; AA, acetic acid; ADF, acid detergent fiber; NDF, neutral detergent fiber; HC, hemicellulose; AN, ammonia nitrogen; AB, aerobic bacteria; LAB, lactic acid bacteria. JCK stands for commercial microbial agent control group; R stands for *Lactiplantibacillus pentosus* alone; LPL stands for combination of *Lactiplantibacillus plantarum*, *Pediococcus pentosaceus*, and *Leuconostoc mesenteroides* subsp. *mesenteroides*; LPLR stands for combination of LPL and *Lactiplantibacillus pentosus*.

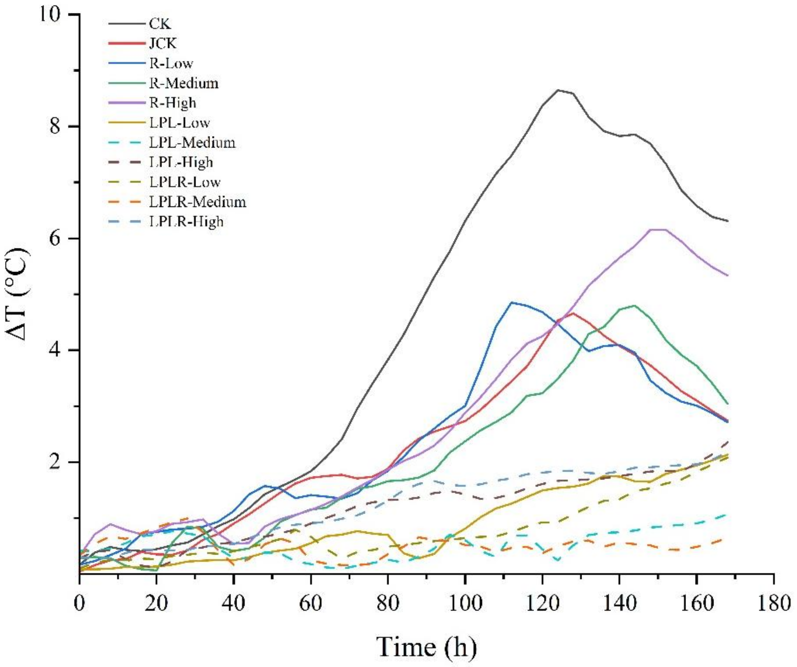

Figure S2. Aerobic stability of corn stover silages exposed to air after 60 d of fermentation

JCK stands for commercial microbial agent control group; R stands for *Lactiplantibacillus pentosus* alone; LPL stands for combination of *Lactiplantibacillus plantarum*, *Pediococcus pentosaceus*, and *Leuconostoc mesenteroides* subsp. *mesenteroides*; LPLR stands for combination of LPL and *Lactiplantibacillus pentosus*. The low, medium, and high concentrations represent 1×10^6, 1×10^7, and 1×10^8 CFU/g FW, respectively.
